# Corona Virus Disease 2019 (COVID-19) as a System-Level Infectious Disease With Distinct Sex Disparities

**DOI:** 10.3389/fimmu.2021.778913

**Published:** 2021-11-29

**Authors:** Modjtaba Emadi-Baygi, Mahsa Ehsanifard, Najmeh Afrashtehpour, Mahnaz Norouzi, Zahra Joz-Abbasalian

**Affiliations:** ^1^ Department of Genetics, Faculty of Basic Sciences, Shahrekord University, Shahrekord, Iran; ^2^ Department of Research and Development, Erythrogen Medical Genetics Lab, Isfahan, Iran; ^3^ Clinical Laboratory, Sina Hospital, Tehran University of Medical Sciences, Tehran, Iran

**Keywords:** COVID-19, interconnectedness, immunity, coagulation, thromboembolism

## Abstract

The current global pandemic of the Severe Acute Respiratory Syndrome CoronaVirus 2 (SARS-CoV-2) causing COVID-19, has infected millions of people and continues to pose a threat to many more. Angiotensin-Converting Enzyme 2 (ACE2) is an important player of the Renin-Angiotensin System (RAS) expressed on the surface of the lung, heart, kidney, neurons, and endothelial cells, which mediates SARS-CoV-2 entry into the host cells. The cytokine storms of COVID-19 arise from the large recruitment of immune cells because of the dis-synchronized hyperactive immune system, lead to many abnormalities including hyper-inflammation, endotheliopathy, and hypercoagulability that produce multi-organ dysfunction and increased the risk of arterial and venous thrombosis resulting in more severe illness and mortality. We discuss the aberrated interconnectedness and forthcoming crosstalks between immunity, the endothelium, and coagulation, as well as how sex disparities affect the severity and outcome of COVID-19 and harm men especially. Further, our conceptual framework may help to explain why persistent symptoms, such as reduced physical fitness and fatigue during long COVID, may be rooted in the clotting system.

## Introduction

Corona Virus Disease 2019 (COVID-19) is an infectious disease caused by SARS-CoV-2, an RNA virus with a crown-like appearance, and spreads rapidly all over the world. It transmits from human-to-human mainly *via* respiratory system (1). COVID-19, known to be a heterogeneous disease that manifests a varying range of symptoms from asymptomatic to severe disease. As an RNA virus, SARS-CoV-2 is a highly mutable virus with a rapidly evolving rate that leads to a various subtypes of the virus. Some characteristics of the SARS-CoV-2 have changed significantly in some evolved subtypes of the virus. Rapid evolution of the virus creates critical changes in SARS-CoV-2 behaviors, including an altered transmission or more severe disease (2). Considering the high rate of alterations in SARS-CoV-2 genome that cause various types of clinical and laboratory manifestations that in turn leads to the progression of COVID-19 towards severe and fatal forms of the disease in some cases, it calls for an urgent need for the identification of aberrated interconnectedness of biological levels of organization of humans and crosstalks between pathways that establish a malicious circuitry involving in COVID-19 pathogenesis. Interconnected cell signaling pathways (interconnectedness) may effectively and precisely transmit innumerable diverse signals, despite an intrinsic potential for improper levels of cross-talk. Network-level mechanisms insulate pathways from crosstalk and allow cells to process information from their environment and respond in ways to input signals. Evolutionarily, metazoan signaling networks are intricate, with incredible levels of crosstalk between pathways where proteins shared between two pathways provoke one pathway’s activity to be modulated by the activity of another. Indeed, crosstalk allows different cell types, each expressing a specific subset of signaling proteins, to trigger distinct outputs when dealt with the same inputs, reacting distinctly to the same environment. To point out, the tissue-specific networks mainly respond distinctly to the inhibition of individual proteins. These findings imply that the intricate interaction between network topology and gene expression that allows various cell types to respond distinctly to the same signals has significant implications for the development of drugs that target signaling processes (3, 4). As COVID-19 clinical manifestations indicate the symptoms, severity of the disease, and corresponding care settings, vary amongst the infected patients (5). One crucial determining factor in COVID-19 severity is the interaction between the virus and the host cells. It is known that the Spike (S) protein of CoronaViruses (CoVs) mediates the binding of the virus to the cell receptors and enables the fusion of the virus with the host cell membrane (6). Of particular interest, SARS-CoV-2 interacts with the RAS *via* ACE2, which was also identified as a functional receptor for Severe Acute Respiratory Syndrome CoronaVirus (SARS-CoV) (7–9). It has been indicated that RAS activation during SARS-CoV-2 infection leads to a number of unfavorable effects, which include vasoconstriction and hypertension, cellular differentiation and growth, endothelial dysfunction, and the formation of Reactive Oxidative Species (ROS) that may ultimately lead to organ damage (10, 11).

In human, ACE2 is expressed in nearly all human organs, such as the upper respiratory tract, alveolar epithelial cells, vascular endothelial cells and macrophages. In addition to acting as the receptor for SARS-CoV-2, ACE2 is a component of RAS which regulates several pathological processes like fibrosis, inflammation, oxidative stress and vasoconstriction (12, 13). Currently, there is a lack of evidence on how aberrated interconnectedness of RAS with the other systems contributed in COVID-19 pathophysiology. It has been hypothesized that the RAS may be involved in the COVID-19 pathogenesis *via* activation of the classic pathway. The broad distribution of ACE2 receptors in the endothelium could potentially allow for widespread effects outside the lung, following SARS-CoV-2 infection (14).

COVID-19 appears as asymptomatic disease or shows mild symptoms in the majority of patients (about 80%) (15, 16). In clinical evaluation, fever, cough, dyspnea, myalgia, and fatigue are the most common symptoms among mildly symptomatic patients. Moreover, uncommon symptoms, including headache, sputum production, hemoptysis, and diarrhea, have been reported in SARS-CoV-2 infection (17, 18). However, the remained proportion of the patients experience severe complications within a short time after infection, such as Acute Respiratory Distress Syndrome (ARDS), Disseminated Intravascular Coagulation (DIC), sepsis followed by organ failure, and death (19, 20). Notably, studies reported elder people are at a greater risk for developing severe forms of COVID-19, and a higher mortality rate was reported among older adults (21). COVID-19 is a complicated multi-system disease that greatly affects the vascular system and hemostasis maintenance. The correlation between the immune system dysfunction and impairment of general hemostasis is well-known and has been addressed in different contexts (1). The innate immune response is the first-line defense of the human body and has a determinant role in protective and/or destructive responses to any infections (22). An effective immune response upon viral infection includes type I InterFeroN (IFN-I) responses and its downstream cascades (23). In SARS-CoV-2, at the first contact with the respiratory mucosa and following SARS-CoV-2 entrance, the production of structural and non-structural proteins of the virus is triggered, subsequently blocking of the interferon response is promoted *via* SARS-CoV-2 N-protein (24). Failed IFN-I response curtails the early viral control and induced the penetration of hyper-inflammatory neutrophils, monocytes and macrophages, which lead to extensive production of pro-inflammatory cytokines and may evoke a cytokine storm that is correlated to the severe manifestations of COVID-19 (25–27).

Clinical evidence of severe patients of COVID-19 indicate that hyper-inflammation, and particular forms of vasculopathy, including Thrombotic MicroAngiopathy (TMA) and intravascular coagulopathy, are frequent features among them. In these cases, an uncontrolled increase of inflammatory cytokines induces vascular hyperpermeability and Multi-Organ Dysfunction Syndrome (MODS) leading to cardiac, hepatic, renal systems’ failure, and eventually death (1). Consistent with a state of hypercoagulability linked with a severe inflammatory response, coagulopathy is an important pathophysiological feature of COVID-19, characterized by the elevated fibrinogen levels, Von Willebrand Factor (VWF), and the fibrin degradation product (D-dimer, a fibrin degradation product that its increases are frequently reported in COVID-19 patients) (28). In fact, a failure to retain hemostasis due to pulmonary injury and MODS creates a critical condition in COVID-19 severe patients (29). A more complicated situation has been reported in the presence of coagulopathy, which is rather a prothrombotic character with a high chance of Venous ThromboEmbolism (VTE) among COVID-19 patients in Intensive Care Units (ICUs) (30). It seems that endothelial dysfunction, microvascular thrombosis, and occlusion, or autoimmune mechanisms may contribute to developing coagulopathy in severe SARS-CoV-2 pneumonia (31). In general, COVID-19 severity is not limited to the respiratory tract and shows age and sex tendencies. Besides the age bias, sex bias with higher numbers of cases, greater disease severity, and higher death rates among men compared to women is one of the interesting features of this disease that might occur due to male-specific factors that increase men’s susceptibility to the SARS-CoV-2 infection (32, 33). A growing body of evidence implies that we need to consider COVID-19 as a system-level infectious disease in which aberrated interconnectedness of immune system dysfunction, aberrant inflammatory responses, and prothrombotic conditions and crosstalks between some pathways occurred during COVID-19 pathogenesis that lead to the various manifestations and severity of the disease. Interconnected biological levels of organization of humans are capable of transmiting a multitude of different signals efficiently and accurately, regardless of a built-in potential for unenviable levels of crosstalks (28, 34, 35). With this in mind, we aimed to throw more light on pivotal aberrated interconnectedness of biological levels of organizations and pathways crosstalks in progression of COVID-19, to provide a well-defined insight to recognize COVID-19-associated pathophysiology as a system-level infectious disease.

## The Role of Renin-Angiotensin System

When SARS-CoV-2 infects cells expressing the surface receptors ACE2 and transmembrane serine protease 2 (TMPRSS2), the active replication and release of the virus causes the host cell to undergo pyroptosis, an inflammatory cell death, and release Damage-Associated Molecular Patterns (DAMPs). In contrast to Angiotensin-Converting Enzyme (ACE), a zinc metalloproteinase and a key regulator of the RAS, ACE2 is an enzyme identified in rodents and humans with a more restricted distribution than ACE (36). In humans, ACE2 is mainly expressed in lung epithelial cells, enterocytes, arterial endothelial cells, and smooth muscle cells in cardiovascular tissues (37). In ACE2/TMPRSS2 dependent cell entry, S protein of SARS-CoV-2 binds to ACE2 to initiate virus entry (38, 39). Once bound, TMPRSS2 cleaves the S protein to allow for membrane fusion. Upon endocytosis, the viral RNA genome is released into the cytoplasm of the host by fusion of the virions with the endosomal membrane (6). This process is the most decisive phase in defining host compatibility and transmissibility of SARS-CoV-2.

The RAS has two pathways, the ACE-dependent pathway (vasoconstrictive side) and the ACE-independent pathway (vasodilative side). Both pathways begin with renin, which is produced by the kidney, converting angiotensinogen from the liver into ANGiotensin I (ANG I). In the ACE-dependent pathway, ANG I is converted to Angiotensin II (ANG II) by ACE. ANG II attaches to its receptor, ANG II Type 1 Receptor (AT1R). This increases Blood Pressure (BP) by causing vasoconstriction and sodium retention. Notwithstanding, in ACE-independent pathway a different enzyme, ACE2, converts ANG I to Angiotensin-1-9 (ANG-1-9) and ANG II to Angiotensin-1-7 (ANG-1-7). ANG-1-7 interacts with two different receptors, Mas and ANG II Type 2 Receptor (AT2R) receptor (40, 41). This pathway works to oppose the actions of the ACE-dependent pathway by causing vasodilation, thereupon lowering BP and providing other cardioprotective effects (40).

**Figure 1:**
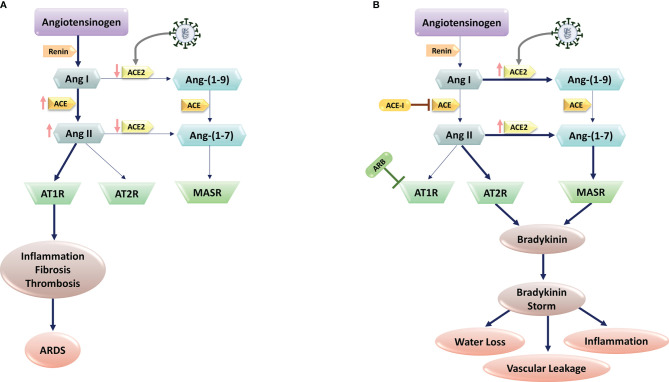
SARS-CoV-2 and Renin-Angiotensin System (RAS) interactions. **(A)** SARS-CoV-2 infection is mediated *via* the human Angiotensin-Converting Enzyme 2 (ACE2), which would cause ACE2 downregulation. Subsequent block of Ang II/Ang–(1-7) metabolism lead to the high levels of Ang II production through ACE upregulation. The imbalanced Ang II/Ang 1–7 causes the suppression of AT2 and Mas receptors with anti-inflammatory properties. In contrast, Ang II and its receptor AT1R activations induce pro-inflammatory effects, fibrosis, and thrombosis, leading to Acute Respiratory Distress Syndrome (ARDS) in lungs. **(B)** In patients with comorbidities, including hypertension, administration of RAS blockers (e.g., ACE-I, and ARBs) suppresses the ACE pathway. In these patients, SARS-CoV-2 infection increase ACE2 expression. Upregulated ACE-2 converts Ang I to Ang–(1-9) and Ang–(1-7), activating the bradykinin pathway *via* MAS and AT2R receptors. Upregulated bradykinin (bradykinin storm) in the lungs of COVID-19 patients would increase SARS-CoV-2 virulence by inducing hypotension, vascular permeability, water loss, and inflammation.

As SARS-CoV infection on lung cells leads to the decreased levels of ACE2 (42), it is postulated that SARS-CoV-2 works in the same vein in individuals without pre-existing conditions to reduce ACE2 expression in lung tissue. Markedly, ACE2 is highly expressed in the lung parenchyma, especially in type II pneumocytes (Alveolar Type II (AT2) cells) (43). Type II cells synthesize and release pulmonary surfactant, enriched with a rather unique phospholipid and four surfactant-associated proteins, which is necessary to maintain alveolar structure (44). Furthermore, Type II cells can differentiate to become Alveolar Type I (AT1) cells, a mechanism for replacement of type I cells that are damaged. The SARS-CoV-2 and SARS-CoV-1 viruses perturb alveoli to cause the main pathology in the lung, with increased fluid entry, cell death and inflammation along with reduction in gas exchange and levels of surfactant (45, 46). Moreover, viral infection triggers ACE2 endocytosis, leading to reduced cell surface expression of ACE2 (**Figure 1A**). Conversely, in individuals with comorbidities including diabetes, cardiovascular disease and hypertension, the ACE independent pathway is activated after SARS-CoV-2 infection due to the administrated drugs in these patients that block RAS. Indeed, pharmacological research concerning the SARS-Cov-2 patients and animal studies revealed frequent use of ACE inhibitor and/or Angiotensin II type I receptor blockers lead to a significant increase in ACE2 expression due to rise of angiotensin- (1–7) levels and decline in Ang-II levels in the plasma (47, 48) (**Figure 1B**). In addition, Gottschalk G et al. reported that ACE2 receptor was strongly upregulated in lungs during SARS-CoV-2 infection. As a result, ACE2 is seen as a double-edged sword in which in normal people the activation of ACE-dependent pathway upon SARS-CoV-2 infection leads to reduced expression of ACE2 that in turn results in ARDS and endothelial dysfunction, while in individuals with comorbidities leads to coagulation, inflammation, and damage to lungs and brain (49–51).

Using RNA sequencing of BronchoAlveolar Lavage (BAL) samples from patients with severe COVID-19 and comparing the results with control samples, Garvin et al. provided further detail into dysregulation of the RAS (52). Of note, the BAL fluid showed profound dysregulation of the RAS. Angiotensinogen and renin increased significantly. The degradation of Inhibitor of nuclear factor Kappa-B Kinase subunit gamma (IKK- γ) by the virus-encoded protein results in blocking production of interferon and ACE transcription. Without ACE, and thereby ANG II, ACE2 is upregulated in the lavage samples that in turn provide exceptionally more entry points for the virus. Furthermore, ACE2 converts ANG I to the fragment ANG-1-9. This fragment activates bradykinin receptor signaling that leads to bradykinin storm (52). In the lavage samples, bradykinin receptors 1 and bradykinin receptors 2 were substantially upregulated (53). Meanwhile, because of the reduction of ACE2 expression in response to SARS-CoV-2 infection ostensively in normal individuals, Ang II/AT1 activation *via* ACE-dependent pathway results in inflammation through an increase in ROS level that in turn leads to a rise in InterLeukin 6 (IL-6) and C-reactive protein levels. Furthermore, Ang II/AT1 activation results in endothelial dysfunction, and in the context of a viral infection, increased endothelial signaling may be the catalyst for initiation of the coagulation cascade in certain individuals. In this pathway, Ang2/AT1 activation results in multiplicative effects on vasoconstriction, BP, endothelial dysfunction, ROS formation, and finally damage to organs (**Figure 1**) (54).

Considering the role of RAS in COVID-19 pathogenesis, the key mechanisms associated with lower COVID-19 severity and mortality in women are: I) Decreased ACE2 methylation in women, resulting in increased ACE2 expression; II) ACE2 is on the short arm of the X chromosome, where up to 30% of genes undergo X inactivation Escape; III) estrogen promotes ACE2 expression. Higher levels of ACE2 could supply a better source to protect tissue after viral entry. Evidence shows ACE2 plays a protective role in chronic pathologies including hypertension, cardiovascular diseases, and acute respiratory distress syndrome, which are the comorbidities representing the risk of worse prognosis in COVID-19. Studies in mouse models support the protective role of ACE2 by showing more severe lung failure upon ACE2 down-regulation that results in overactivation of the Angiotensin (Ang) II/AT1R axis that may explain the multi-organ dysfunction seen in patients (**Figure 1**) (55, 56). However, low estrogens in men lead to the absence of higher levels of ACE2, supporting the ACE pathway in the RAS axis that facilitates disease severity in men with the same viral load as women. In men, androgens increased the expression of TMPRSS2 that supports viral entry, resulting in male increased susceptibility. In contrast, decreased levels of androgens in women may keep TMPRSS2 expression at lower levels, providing an additional defensive determinant against the development of COVID-19 infection (57).

## Immune System and Inflammatory Responses

The human immune system protects us against life-threatening and pathogenic agents. As the first line of immune defense, the innate immune system plays an indispensable role against viruses (58, 59). Germline-encoded Pattern Recognition Receptors (PRRs) are essential immune receptors that trigger antiviral innate immune responses through sensing the conservative structures of viruses. PRRs have a crucial role in immune responses by recognizing Pathogen-Associated Molecular Patterns (PAMPs) that are unique microbial molecules and DAMPs that are self-derived molecules elicited from damaged cells (60, 61). Two PRRs, Toll-Like Receptors (TLRs) and Retinoic acid-Inducible Gene-I [RIG-I Like Receptors (RLRs)], are identified to have a crucial function in sensing viral ssRNA genome and dsRNA replication intermediates. TLRs and RLRs recognize these nucleic acid species and bind certain intracellular adaptor proteins, which activate NF-κB, mitogen-activated protein kinases, and interferon regulatory factors, which control the transcription of genes encoding IFN-I and other inflammatory cytokines, which are critical for virus elimination (62).

**Figure 2:**
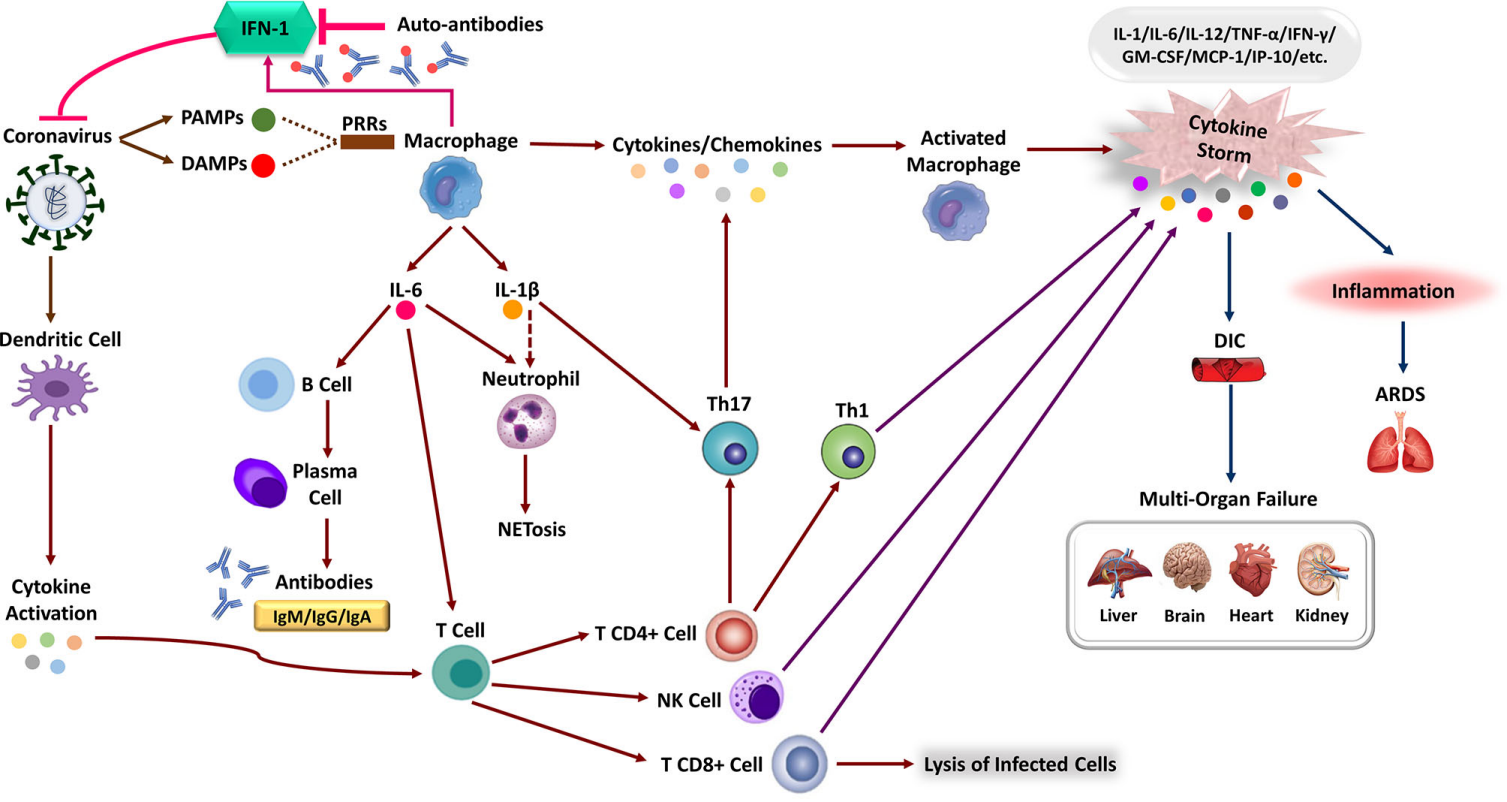
The immune response corporation in covid-19 disease. Type 1 interferon (IFN-I) immune response plays a pivotal role in effective immunity against SARS-CoV-2 infection and rapid viral clearance. Following SARS-CoV-2 infection, the IFN-1 initiates *via* recognizing PAMPs/DAMPs by PRRs of the human immune cells and releasing inflammatory cytokines (e.g., IL-1 beta, IL-6). The expression of numerous inflammatory cytokines leads to activation of neutrophils (NET formation) and attraction of different immune cells toward the site of infection. Macrophages activation, Dendritic Cell (DC) maturation and inflammatory cytokines stimulate the adaptive immune response to join the fight against SARS-CoV-2. Activation of T-cells *via* cytokines promotes multiple T-cell differentiation (i.e., CD4 +, CD8 +, NK cell, Th1) that directly kills virus-infected cells. Activated B-cells produce virus-specific antibodies that would participate in a successive immune response. In patients with severe forms of COVID-19, the delayed IFN-I pathways or neutralizing auto-Abs will lead to immune system overreaction and the generation of high inflammatory cytokine levels. The aberrant induction of the immune system and the production of various pro-inflammatory cytokines (e.g., IL-1, IL-6, MCP-1, TNF-α, and etc.), the so-called “cytokine storm,” leads to severe COVID-19 immunopathology. These can cause severe local damage to the lungs (e.g., ARDS) and other organs (e.g., DIC), and in the worst case, can lead to Multi-Organ Failures (MOF) and even death.

PRRs can identify SARS-CoV-2, like other RNA viruses, leading to the activation of IFN-I response (29, 63, 64). To point out, multiple viral structural and non-structural proteins of SARS-CoV impair IFN-I response, resulting in a hyper-inflammatory response. To enumerate, SARS-CoV is able to antagonize the TLR signaling pathway *via* its papain-like protease (65). Indeed, antagonized IFN responses in SARS-CoV-2 infection is likely to occur at various pathways involved in immune responses (**Figure 2**) (1). Like SARS-CoV and Middle East Respiratory Syndrome CoronaVirus (MERS-CoV), vesicle-dependent replication of SARS-CoV-2 genome may cause protecting RNA genome from host detection by cytosolic (e.g., RIG-I) and endosomal (e.g., TLR3/7) PRRs (66, 67). Furthermore, it is possible that INF-I inhibitions cause the antiviral response delay (e.g., by inhibiting TLR3 and TLR7 signaling pathways, virus-encoded antagonists to the IFN responses and auto-antibodies) that in turn it facilitates replication of virus particles and extensive virus cytopathic effects at the early stages of the COVID-19 disease (29, 63, 65, 68–70). Moreover, the infection of ACE2-positive ATII pneumocytes would lead to a significant decrease in the production of pulmonary surfactant and exposing the TLR4. In addition, direct or indirect SARS-CoV-2 binding to TLR4 causes an increase in the expression of ACE2 *via* IFNs and interferon-stimulated genes. Therefore, the virus may directly enter the cell using TLR4 and cause aberrant TLR4 signaling (64). However, Onabajo et al. show that the ACE2 induced by IFN is a truncated isoform of ACE2, deltaACE2, which is nonfunctional in binding the SARS-CoV-2 spike protein (71). Correspondingly, the activity of SARS-CoV-2 non-structural protein 14, which has a (guanine-N7)-methyltransferase activity, results in the efficient escape of viral RNA from detection by the RIG-I receptor. RIG-I is a critical cytosolic RNA sensor that interacts with the mitochondrial antiviral signaling proteins to activate the downstream programs such as type I/III IFN responses (72). This suboptimal innate immune operation by host PRRs during SARS-CoV-2 infection induces non-productive inflammatory responses, resulting in a cytokine storm and disseminated damage to the host. Generally, cytokine storm is a hyperactive immune response characterized by the release of different cytokines, chemokines, and other immune mediators that may hurt host cells. Notably, the majority of cytokine storm mediators demonstrate pleiotropic downstream effects mostly interdependent in their biological activity. Therefore discovering the precise dysregulated inflammatory response implies pathogenesis of the disease has been a major challenge in critical illness like COVID-19 (73). Although reports suggest that like SARS-CoV, SARS-CoV-2 have subversion strategies against innate immune signaling and antiviral interferon response, the exact aberrations of interconnectedness and crosstalks remain poorly understood (66, 72, 74). Commonly, cytokine storm is considered a critical determining factor associated with adverse outcomes of the COVID-19 disease; however, there is variation and sometimes-even discrepancies between studies about the exact detail of this phenomenon. Despite the poorly defined pathophysiology of cytokine storm, widespread acceptance of the term in COVID-19 has motivated to apply potent immunomodulatory therapies such as IL-6 inhibitors and high-dose corticosteroids. Nevertheless,in comparison with the other causes of ARDS (median IL-6 level is 10- to 200-fold higher than COVID-19) the lower levels of circulating cytokines in COVID-19 may not be representative of lung inflammation and need to be determined whether COVID-19 related ARDS phenotype is associated with the cytokine storm (73, 75).

SARS-CoV-2 infection in pulmonary airway epithelial cells triggers local and systemic pathological responses through recruiting macrophages and monocytes, expressing inflammatory cytokines, and inducing adaptive immune responses. Although immune response resolves the infection in most COVID-19 patients, immune system dysfunction leads to hyper-inflammation resulting in a cytokine storm that causes severe lung injury and multi-system damages (1, 76, 77). These pro-inflammatory cytokines stimulate an influx of neutrophils and other myeloid cells into the lung, creating a local hyper-inflammatory response and significant immunopathology (78). The association of hyper-inflammatory response with lung injury, high rate of progression to ARDS, Multi-Organ Failure (MOF), and unfavorable prognosis of severe COVID-19 has been described in several studies (15, 79, 80). In the same way, overproduction of pro-inflammatory cytokines (Tumor Necrosis Factor alpha (TNF-α), IL-1, IL-6, and IL-1β), monocytes, and neutrophils, followed by a sharp decrease in lymphocytes, have been shown in various studies (15, 45, 81).

Clinical studies show major differences in the expression of inflammatory markers and immune phenotypes between moderate and severe cases, along with disease progression (82). An elevated level of immunomodulatory cytokines, including IL-1α, IL-1β, IFN-α, IL-17A, and IL-12p70, constitute COVID-19 signature that exhibits dynamic features associated with clinical manifestations (**Figure 2**) (15, 45). However, a considerable extent of other cytokines and chemokines, including IFN-γ, thrombopoietin (associated with blood clotting aberrations), IL-1, IL-6, IL-8, IL-2, IL-7, IL-10, Granulocyte Colony-Stimulating Factor (G-CSF), Interferon-inducible Protein 10 (IP-10), Monocyte Chemoattractant Protein 1 (MCP-1), Macrophage Inflammatory Protein 1α (MIP-1α) and TNF, C-X-C Ligand 10 (CXCL10), C-C motif Ligand 2 (CCL2) and CCL3, have been reported in severe patients (15). IL-6 is thought to play a pivotal role in pathology of COVID-19, considering its highest levels in non-survivors and critically ill patients. Notwithstanding that the IL-6 levels in these patients continue to increase over time, the mechanism leading to its elevation in severe COVID-19 is not currently clear. It is possible that aberrant activation of virus-specific PRRs like TLR4 triggers some sorts of crosstalks, which then drives IL-6 production (1, 83, 84).

The high levels of IL-6 observed in COVID-19 patients are apt to elicit Neutrophil Extracellular Traps (NETs) which include extracellular DNA fibers, histones, microbicidal proteins, proteases and oxidant enzymes to be released by neutrophils (**Figure 2**) (85, 86). Pathological effects of NETs have been indicated in propagated inflammation and respiratory failure. Although neutrophils are early indicators of SARS-CoV-2 infection, their excessive recruitment causes an unregulated NETs release that contributed to organ damage and mortality in COVID-19 patients (87). A significant increase in the blood neutrophil counts has been indicated in the COVID-19 patients admitted to the ICU. Consistent with SARS-CoV and MERS-CoV, increased levels of pro-inflammatory cytokines may drive an influx of neutrophils and other myeloid cells into the lung. The elevated neutrophil/lymphocyte ratio is considered an early prognostic marker in SARS-CoV-2 infection, which increases in parallel with the severity of the disease. Transcriptional analysis of BAL fluid and peripheral blood mononuclear cells showed that elevated CXCL2 and CXCL8 chemokines contribute to neutrophils recruitment and aggravate the inflammatory response in COVID-19 patients (66, 76). Of note, besides neutrophils, inflammatory chemokines cause the recruitment of other innate immune cells like monocytes, Dendritic Cells (DCs), and Natural Killer (NK) cells. These innate immune cells respond to tissue damage *via* producing several cytokines, including IL-1, IL-6, and TNF. Consequently, various immune cells such as neutrophils, macrophages, and T cells mobilize from the blood circulation into the infected tissue that leads to diffuse alveolar damage, capillary damage, vascular barrier damage, MOF and ultimately death (88–90).

Considering that the association of COVID-19 severity with aberrant inflammatory response has been demonstrated in various studies, the exact molecular mechanisms causing innate immune response dysregulation are yet to be elucidated (1). In fact, activation of innate immunity against pathogens would benefit the host in two ways. First, effective initiation of innate immune responses would directly kill pathogens. If those responses failed to eliminate the infection, the adaptive immune responses would initiate to provide a second layer of immune protection (61). In SARS-CoV-2 infection, however, inadequate and delayed intracellular innate immune responses failed to prime adaptive immune response for a long time, leading to severe lung disease and MODS. This particular condition in SARS-CoV-2 infection leads to a dis-synchronized innate and adaptive immune response (74), a kind of aberrated interconnedness.

In general, the high specificity of the adaptive immune system enables a highly regulated and targeted immune response to eliminate the infected cells and neutralizing free virions (91, 92). The effective adaptive immune response contains both humoral (B-cells) and cellular (T-cells) responses. The activity of CD8+ T cells and B cells is predominantly regulated by CD4+ T cells. In contrast, the targeted killing of virus-infected cells is accomplished by CD8+ T cells. B cells Antibodies (Abs) block surface proteins and agglutinate virions and thereby prevent infection (**Figure 2**) (93). Humoral immune system produces specific Abs that are expected to neutralize different antigens, limit virus viability and also elicit T-cells to the location of infection by delivering them. But some produced Abs against CoVs result in more complicated situations. Neutralizing antigen-specific Abs against CoVs is mostly targeting spike domain (94). A group of anti-spike Immunoglobulin G (IgG) in acute phase of infection before viral clearance can end in some inflammatory hyper-reactions (95). This phenomenon is due to a shift in polarization of alveolar macrophages and their related cytokines to a pro-inflammatory state. This alteration is mostly because of the binding affinity of anti-spike IgG and receptors presented on macrophages (91). Equally important, some infected individuals with SARS-CoV-2 exhibit a range of B cell population producing auto-antibodies similar to those observed in Systemic Lupus Erythromatose (SLE) (96). These Abs are mostly antinuclear Abs, rheumatoid factor (anti-IgG-Fc Abs), AntiPhosphoLipid Antibodies (APLAbs), and Abs against IFN-I. These Abs suppress the innate immune response *via* IFN-I antagonizing and directly contributes to pathophysiology of COVID-19 (97). Furthermore, auto-antibodies target immune-related proteins including those involved in lymphocyte function and activation, leukocyte trafficking, the type I and type III IFN responses, type II immunity and the acute phase response increase in patients with severe COVID-19. With this in mind, patients with auto-antibodies against IFN-1 experience extended durations of hospital admission due to impairment of virological clearance (98).

Equally important, like SARS-CoV and MERS-CoV infection, a drastic diminish in the number of T cells would ultimately cause an inefficient T cell response in severe COVID-19 patients (99). The critical role of CD4+ and CD8+ T cells in virus clearance has been demonstrated in the immunodeficient mouse model of SARS-CoV (100). Considering the pivotal function of T cell immunity in SARS-CoV infection, T cells appear to have a protective role against SARS-CoV-2 infection. However, the function of T cells in the resolution, and long-term protection against SARS-CoV-2 remains debated. The significant dysregulation of T-cell response has been indicated in severe COVID-19 patients; However, it has not been clear which T-cell activities contribute to the development of the disease severity (101, 102). Lymphopenia is one of the prominent features of severe COVID-19 that have been reported to affect CD4+, CD8+, B, and NK cells in some patients. Coupled with the high IL-6, IL-10, and TNF levels, lymphopenia was observed in severe COVID-19 manifestations (103–105). In particular, different studies have demonstrated that lymphopenia harbors T cells biased in COVID-19, which would lead to hyperactivation of these cells (101). While activation of T cells seems to have positive effects, SARS-CoV-2 may have mechanisms to restrict T cell activation. Regarding the peripheral lymphopenia observed in patients with COVID-19, the autopsy studies showed that the lymphocytic infiltration to the respiratory tract or adhesion to inflamed respiratory vascular endothelium is not excessive (106, 107). Observed lymphopenia in adult COVID-19 patients is possibly multifactorial (108). Lymphopenia in patients with severe disease may be related to cytokine over-production possibly by a direct effect of the cytokines on T cell populations (109, 110) and/or indirect effects through other cell types including dendritic (111) cells and neutrophils (112, 113). Augmented expression of pro-apoptotic molecules (apoptotic loss) (114), probably decreased mobilization of lymphocytes from bone marrow, and immunosenescence may also partake in T cell depletion (108, 115). Moreover, with unknown mechanisms, limited MHC I and MHC II antigen presentation was demonstrated in association with T cell dysregulation in these patients (116). In contrast to the mild SARS-CoV-2 infection that harbors a successful lymphocyte-mediated virus clearance, T cells are functionally exhausted, a state that arises during many chronic infections (117), and express a high level of Programmed cell Death protein 1 (PD-1) and T-cell immunoglobulin mucin-3 in severe COVID-19 patients (117, 118). Besides, the implication of the expression of these markers in acute viral infection relates rather with the activation state than with functional exhaustion (119). Moreover, the observed decrease in IFN-γ and IL-21 production would support CD8+ and CD4+ T exhaustion in SARS-CoV-2 patients. In addition, the functional exhaustion of NK cells has been demonstrated in the persistence COVID-19 condition as seen in cancer and chronic viral infection (99, 120). Nevertheless, this exhaustion is likely to be transient, as the decreased expression of PD-1 has been observed in recovered patients from ICU compared to the severe ICU ones (121).

**Figure 3:**
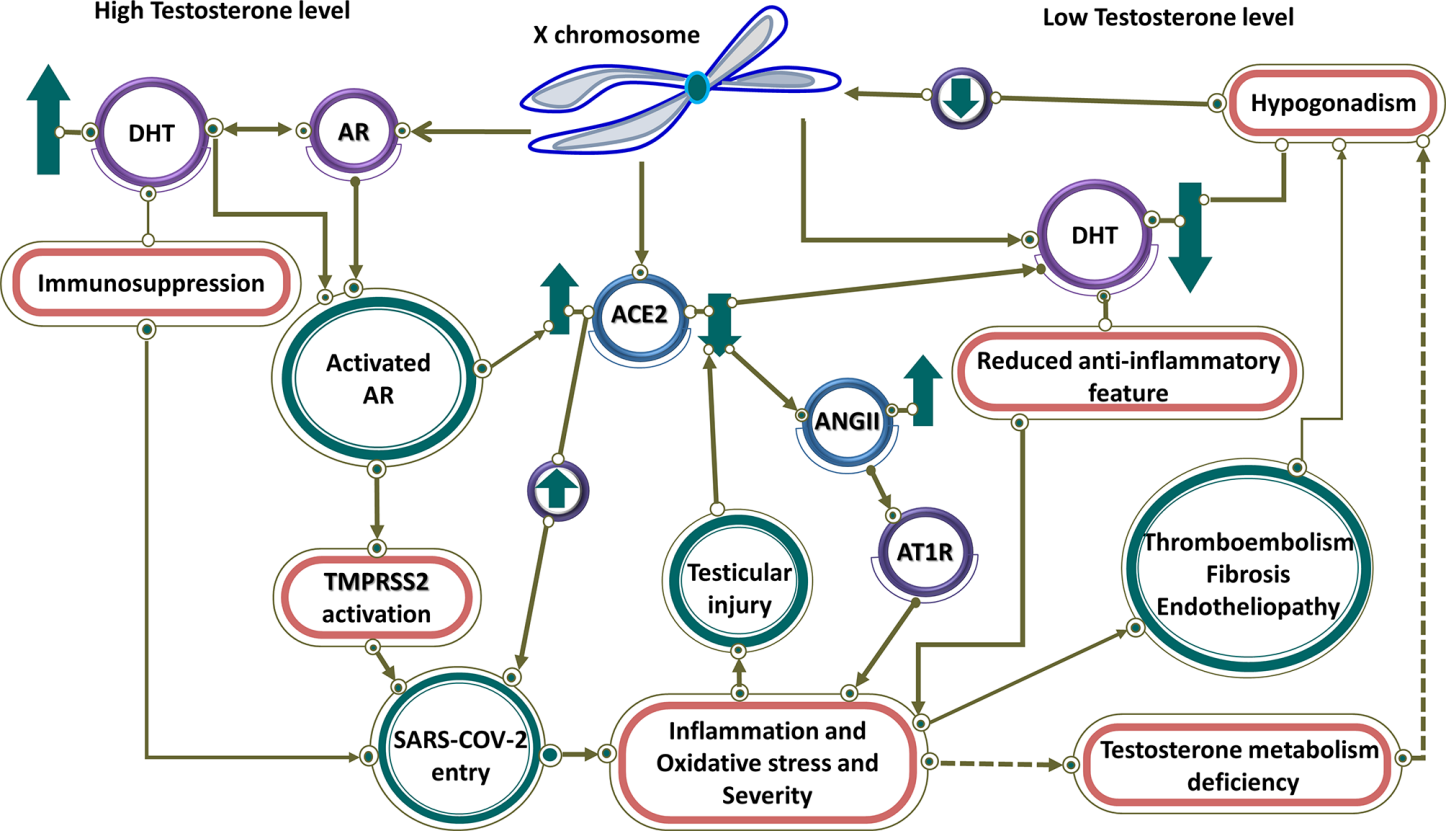
High and low testosterone impact on COVID-19 severity. Controlled by androgen, both TMPRSS2 and ACE2 are regulated by genes on the X chromosome. At high testosterone levels, both receptors are up-regulated, resulting in SARS-CoV-2 entry. Testosterone has an immunosuppressive feature, which leads to more virus entry and disease severity. In contrast, it has linked low testosterone level caused by hypogonadism in men to increased infection of COVID-19. Therefore, in men with hypogonadism, caused by various factors such as reduction of ACE2, testicular damage, and thromboembolism through hyperinflammation caused by the viral infection, the level of testosterone is reduced and the anti-inflammatory effect of testosterone is blocked, resulting in disease severity. Low testosterone results in downregulation of ACE2 that through activation of AR1T by ANG II leads to inflammation and disease severity.

Eventually, a sex-specific immune transcriptome is documented in humans, resulting in females exhibiting an enhanced adaptive immune response, while males show enhanced aspects of innate immunity (122). Equally important, in the context of the COVID-19, there is a difference in immune responses and in turn in severity between males and females. Male patients had substantial induction of non-classical monocytes and higher plasma levels of innate immune cytokines such as IL-8 and IL-18. By contrast, female patients during SARS-CoV-2 infection had substantial T cell activation compared to male patients. In particular, poor T cell response was exclusively associated with worse prognosis in male patients, which negatively correlated with patients’ age. By contrast, higher levels of innate immune cytokines were associated with worse disease progression in female patients, but not in male patients (123). In general, since an extra X chromosome exists in women, cellular mosaicism created by X inactivation in females may contribute partly to the more efficient immune response against SARS-CoV-2. Besides, more ICU admissions and mortality rates are observed in men with COVID-19. To point out, there are many X-linked genes that are related to the immune system, including PRR (for instance TLR7 and TLR8) and the major TLR signaling regulators (like IL-1 receptor-associated kinase 1), that may have a higher copy number in females (124). To emphasize, the biallelic expression of TLR7, an endosomal innate immune receptor, through X chromosome, may potentially cause a more substantial IFN-I response in the early stages of COVID-19 in female patients. Furthermore, increased IFN-α production triggered by TLR7 ligands as shown by *in vitro* experimental observations are detected more in females and subsequently lead to a more rapid antiviral response (29, 124). Moreover, Female reproductive steroids are anti-inflammatory, reshape competence of immune cells, stimulate Ab production, and promote proliferation and repair of respiratory epithelial cells, suggesting they may protect against COVID-19 symptoms (125). Equally important, steroid hormone receptors play a pivotal role in sex-dependent immunity, as defined by lower T lymphocytes percentage in men compared to women. Meanwhile, multiple pieces of evidence imply that there are associations between testosterone levels (normal male level vs age-related hypogonadism) in COVID-19 pathogenesis, indicating its function as a double-edged sword. Testosterone and dihydrotestosterone mediate their actions *via* the Androgen Receptor (AR), a ligand-dependent nuclear transcription factor (126). High testosterone impact on COVID-19 severity through the TMPRSS2 Connection (**Figure 3**) (127). Taken together, epidemiological data emerging from the COVID-19 pandemic, backed by animal studies and further by preliminary clinical studies in diverse clinical settings, support the notion that high testosterone levels acting *via* the AR attune TMPRSS2 function positively to enhance SARS-CoV-2 S proteins and eventually increase COVID-19 infectivity and severity. Additionally, AR mutations or other gene polymorphisms along the pathway of SARS-CoV-2 pathogenesis may further lead to COVID-19 progression and deterioration. This concept ought to be further explored in future studies (127). However, low testosterone, a characteristic biomarker of aging males with functional hypogonadism, impact on COVID-19 severity through the ACE2 Connection (**Figure 3**) (127). To clarify, in COVID-19, SARS-CoV-2 infection may impact the testes *via* binding to ACE2 expressed in the Sertoli and Leydig cells, provoking infertility and inhibiting testosterone production (128). It emphasized that low testosterone serum level is associated with SARS-CoV-2 infections and COVID-19 severity in critically ill patients through lower immunomodulatory properties of androgen antiviral effects (129). Altogether, low testosterone levels appear to be a major factor for poor prognosis and mortality in COVID-19 male patients. This is likely to be significantly exacerbated in men with co-morbid conditions admitted to the ICU. Further research is needed to prove this concept (127).

By all means, COVID-19 is a complicated multi-system disease that greatly affects the immune system and homeostasis maintenance of the body (**Figure 2**) (130, 131). A serious clinical implication and involvement of aberrated interconnectedness and possibly triggered crosstalks have been observed in COVID-19 pathogenesis. The correlation between the immune system dysfunction and impairment of homeostasis is well-known and has been addressed in different contexts (132). Clinical evidence indicates that hyper-inflammation, and particular forms of vasculopathy, including TMA and intravascular coagulopathy, are frequent features of COVID-19 among severe patients (29). In these cases, an uncontrolled increase of inflammatory cytokines induces vascular hyperpermeability and MODS, leading to failure of cardiac, hepatic, renal systems, and eventually death (1). In fact, a failure to retain hemostasis due to pulmonary injury and MODS creates a critical condition in COVID-19 patients (29, 130). A more complicated situation has been reported in the presence of coagulopathy, which is rather a prothrombotic character with a high chance of VTE among COVID-19 patients in ICUs (30). As a result, the overproduction of inflammatory cytokines and the over-activation of immune cells during SARS-CoV-2 infection promote endothelial dysfunction and vascular permeability. Further investigations are mandatory to explore possible mechanisms behind these aberrated interconnectedness and possibly triggered crosstalks (29, 133).

## Coagulopathy and endothelial dysfunction

**Figure 4:**
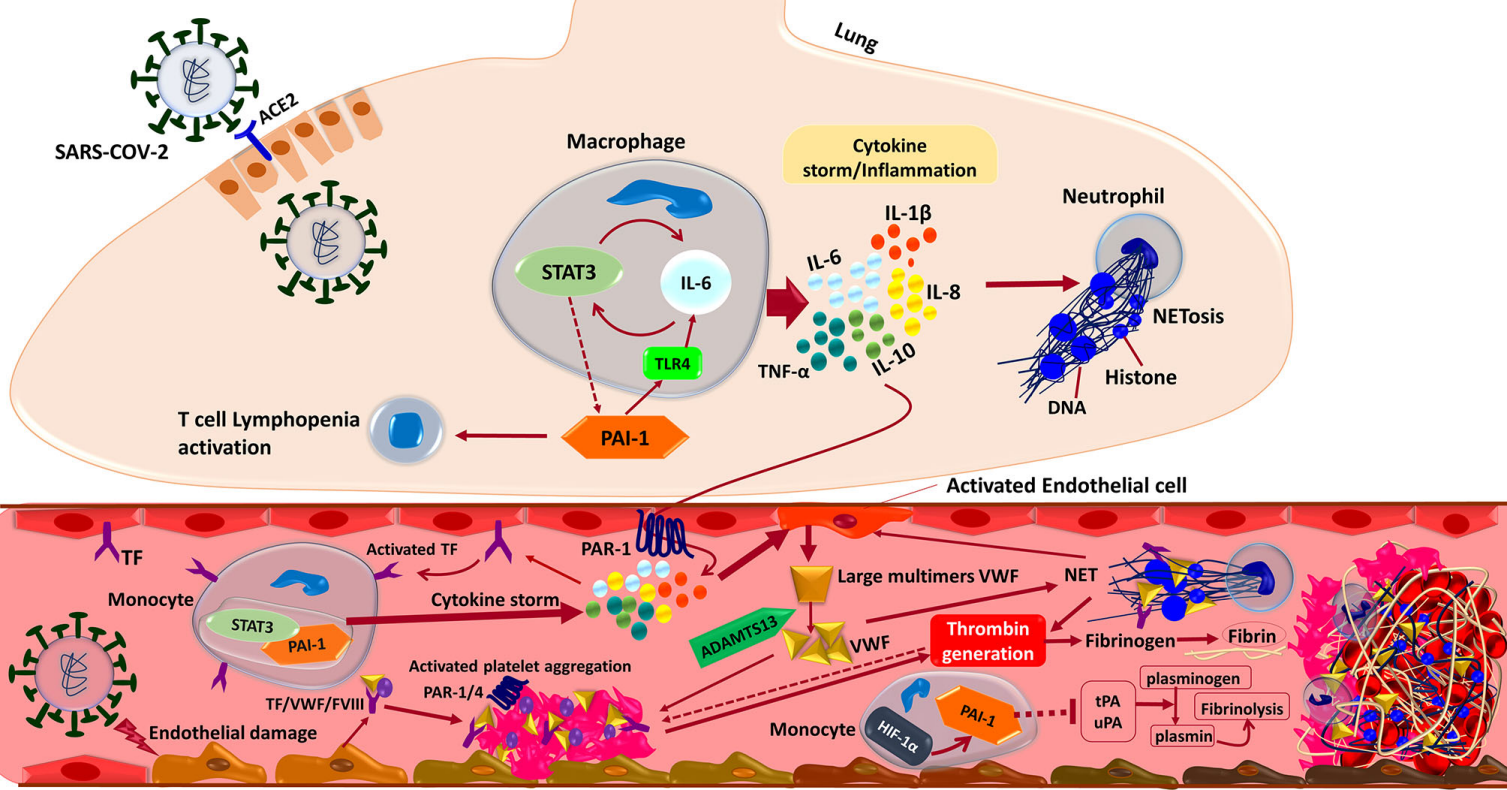
Interactions between the components of the immune system and coagulation cascades that induce thrombosis in COVID-19. In brief, the SARS-CoV-2 infection triggers an inflammatory response in the alveolar lumen by macrophages and neutrophils. Overexpression of PAI-1 and its interaction with TLR4 induce the IL-6 expression and STAT3 activation in the lung of COVID-19 patients. Subsequent production of inflammatory cytokines such as IL-1β, IL-8, IL-6, and TNF-α causes neutrophils to be recruited and release Neutrophil Extracellular Traps (NETs). NETs directly activate factor XII, thereby activating the contact-dependent clotting pathway. The PAR-1 receptor mediates cytokine-dependent Tissue Factor (TF) activation, which stimulates the STAT3/PAI-1 complex in blood’s monocytes and enhances inflammatory cytokine production leading to extrinsic coagulation cascade activation and thrombosis formation. Moreover, inflammatory cytokine in combination with the direct binding of SARS-CoV-2 to the endothelial cells triggers Von Willebrand Factor (VWF) secretion cleaved by ADAMTS13 to the regular size. Activated TF/VWF/FVIII complex in cooperation with NETs recruits platelets to adhere to endothelial surfaces and provoke intrinsic coagulation cascades. Altogether these events ultimately driving thrombin formation from circulating prothrombin, which cleaves fibrinogen to fibrin and stimulates thrombosis formation. Besides, suppression of endothelial enzymes and Plasminogen Activators (tPA/uPA) would aggravate the coagulopathy state by preventing effective fibrinolysis. Both tPA and uPA participate in the normal coagulation-plasmin-fibrin pathway, can be inhibited by PAI-1/HIF-1α in severe COVID-19 patients with ARDS.

Coagulation is a tightly regulated process that involves interactions between numerous blood components called coagulation factors and mechanisms that prevent thrombosis (134, 135). Currently, the so-called ‘COVID-19 associated coagulopathy’, is considered to be a critical player in the pathophysiology of SARS-CoV-2 infection, especially in its severe form (136–138). Coagulation and inflammation are highly integrated and delicately balanced biological systems with extensive crosstalk to optimize the body’s response to any damage and invasion. The impaired interplay of these systems affects both homeostasis and hemostasis, leading to various conditions with varying degrees of excessive inflammation, thrombosis, or bleeding that may lead to tissue damage and MOF (**Figure 4**) (34, 139–141). The hyper-coagulation state in COVID-19 is triggered by the deep and complex inflammatory response to the virus *via* the activation of tissue factors (TFs) on the surface of activated endothelial cells. Bidirectionally, coagulation and inflammation drive an intensifying circle of events by inducing Protease-Activated Receptors (PARs) mediated inflammatory signaling, participation of innate immune pathways, the engaging of the PC–thrombomodulin mechanism as negative regulatory systems, and the playing roles of NETs, and the fibrinolytic system (34, 142). PARs express in many cell types, including immune cells, platelets, endothelial cells, and smooth muscle cells. After proteolytic cleavage by serine proteases such as factor X and thrombi, they activate and induce the production of cytokines and chemokines. PAR-mediated activation of adhesion molecules in endothelial cells results in the production of IL-6 and IL-8 by fibroblasts and monocytes and increased platelet effects *via* a positive feedback loop, amplifying inflammation and procoagulant processes (34, 138, 142).

Mounting evidence shows that coagulopathy events, including immunothrombosis and thromboinflammation, have been observed in COVID-19 (28, 143). Although pieces of literature are controversial and show conflicting findings (144–146), severe COVID-19 patients specially critically ill ones not responding to shock management may show features of systemic hyper-inflammation called Macrophage Activation Syndrome (MAS) or cytokine storm, also known as secondary Hemophagocytic LymphoHistiocytosis (sHLH). It should be noted that the cytokine profile similar to MAS/sHLH has also been observed in COVID-19 patients, especially the increase of IL1β, IL2, IL6, IL17, IL8, TNF and CCL2. Furthermore, like DIC associated with MAS/sHLH, there is evidence that D-dimer levels are elevated in COVID-19 pneumonia which may suggest that the virus-induced hyper-inflammatory pulmonary immunopathology is spreading to the adjacent microcirculation with a broad secondary fibrinolytic activation (**Figure 4**) (144, 147). An increase in D-dimer indicates that COVID-19 patients are in a hypercoagulable state, which can be attributed to the following reasons. First, viral infections are often accompanied by a threatening pro-inflammatory response and inadequate control of the anti-inflammatory response. It can cause endothelial cell dysfunction, leading to excessive thrombin production. Second, the hypoxia found in severe COVID-19 can stimulate thrombosis through both increasing blood viscosity and a hypoxia-inducible transcription factor-dependent signaling pathway. Third, hospitalized severe COVID-19 patients are more likely to have risk factors such as advanced age, underlying diseases, long-term bed rest, and receiving invasive treatment, which are risk factors for hypercoagulability or thrombosis. To point out, the dissection of the lungs of critically ill patients with COVID-19 shows occlusion of small pulmonary vessels and microthrombosis (148). Fourth, some patients may develop septic coagulopathy or even DIC. Elevated D-dimer is always associated with adverse events. In some clinical patients with SARS-CoV-2 infection, acral gangrene in hypercoagulation state has been described pathophysiologically, indicating that coagulation is the most significant cause of vascular thrombosis and venous gangrene in COVID-19 cases (149). Of note, the serological observations of these COVID-19 patients revealed increased D-dimers associated with antithrombin III deficiency, illustrating wide vascular microthrombi and necrosis in skin biopsy (148, 149). Finally, Similar to COVID-19 non-survivors, DIC is one of the major complications reported in fatal MERS-CoV cases. Markedly, a case of MERS-CoV induced DIC, intracerebral hemorrhage, and MOF appeared two weeks’ post-admission in an otherwise stable patient. Furthermore, a fatal case of MERS-CoV was associated with DIC, hyperkalemia, ventricular tachycardia, and cardiac arrest (150–152).

Most compelling evidence shows that endothelial cells can be infected by SARS-CoV-2, leading to endothelialitis. The initiation of inflammation-induced coagulation is mostly mediated by the expression of the TF pathway (CD142). TF is expressed in both mononuclear cells in response to pro-inflammatory cytokines (mainly IL-6) and vascular endothelial cells to promote the conversion of prothrombin into thrombin resulting in fibrin-based blood clots due to the conversion of circulating fibrinogen into fibrin (**Figure 4**) (153–156). To point out, evidence reveals that severe cases of COVID-19 are commonly dependent on a positive feedback loop established between Signal Transducer and Activator of Transcription-3 (STAT3) and Plasminogen Activator Inhibitor-1 (PAI-1) that results in the over-stimulation of the STAT3/PAI-1 signaling network that is shared among diverse disease manifestations and leads to catastrophic consequences (157). To enumerate, PAI-1 is highly expressed in lungs and plasma of COVID-19 patients (158, 159). Furthermore, PAI-1 interacts with TLR4 and triggers the expression of IL-6 that activates STAT3 (157, 158). In turn, STAT3 and PAI-1 augmented thrombosis and coagulopathy in COVID-19 possibly by effectively suppressing urokinase-type Plasminogen Activator (uPA) and tissue-type Plasminogen Activator (tPA) (**Figure 4**) (158–160).

Neutrophils kill and clear invading microorganisms through phagocytosis and degranulation. NETs are a novel antimicrobial strategy of neutrophils that are released from neutrophils into extracellular space to catch and kill invading bacteria to protect the host from infection (161). Regarding the pathophysiology of COVID-19, immunothrombosis and release of NET lead to inflammatory lung/organ damage and thrombosis *via* enhanced NETosis in which neutrophils are more prone to spontaneously form NETs by adopting the low-density phenotype (28, 162–165). Notably, isolated neutrophils from patients with COVID-19 displayed increased NET release at baseline, similar to neutrophils stimulated by phorbol myristate acetate from healthy donors, implying that the environment of the COVID-19 plasma provokes NET formation (**Figure 4**) (28, 165). In the same vein, high levels of NETs detected in the sera of COVID-19 patients can trigger NETs formation *in vitro* in neutrophils collected from healthy volunteers (166, 167). Further, direct stimulation of NETosis by APLAbs of COVID-19 patients (28, 168), as well as the involvement of infectious diseases in the AntiPhospholipid Syndrome (APS) (28, 169), indicates that SARS-CoV-2 infection possibly synergize with APLAbs to provoke the immunothrombotic process. To clarify, Higher APLAbs titers are associated with increased neutrophil and platelet activity and more serious respiratory disease (28, 170). NETs also contribute to acute lung injury by inducing macrophage release of IL-1β, which in turn can reinforce the formation of NET (25, 28, 171). Furthermore, it is known that most of the inflammatory mediators increased in COVID-19 patients regulate the activity of neutrophils through the expression of chemotactic factors (15, 28, 172). Altogether, the evidence indicate that dysregulation of cytokine release might be maintained by NET-mediated crosstalk between neutrophils and macrophages, leading to disordered or disproportionate immunothrombotic status (28).

As a key mediator of hemostasis and plasma glycoprotein, the VWF takes circulating platelets at the vascular injury sites and mediates activation and aggregation of platelets (166, 173). VWF is a multimer that exists in the endothelial Weibel-Palade bodies and releases under the stimulation of cytokines. Proteolytically cleaved by A Disintegrin And Metalloproteinase with ThromboSpondin type 1 motifs, member 13 (ADAMTS13) to regulate the size and activity of VWF multimers, ultra-large VWF multimers are processed into smaller VWF forms to prevent the formation of thrombus (166, 174). To clarify, high molecular weight forms of excessively released VWF may provoke microcirculatory dysfunction through spontaneous binding to platelets resulting in TMA (175, 176). Considering COVID-19, VWF antigen levels (VWF : Ag) and VWF : Ristocetin cofactor activity (VWF : RCo) have significantly increased in moderate and severe cases than in normal controls possibly without change in ADAMTS13 activity (**Figure 4**) (175).

Overall, NETs and VWF/ADAMTS13 axis are essential for thrombosis and inflammation. Given these points, recent evidence shows that the axis is associated with and contributes to the poor prognosis of COVID-19 patients manifested initially by ARDS and then developed to more complex clinical phenotypes, including thrombotic thrombocytopenic purpura-like syndrome, hepatic coagulopathy, MODS, and extensive micro- and macrovascular thrombosis (163, 166, 177). There are increasing global reports considering APLAbs in COVID-19–associated coagulopathy (178). APS is an acquired thrombophilia in which patients develop pathogenic auto-antibodies against phospholipids and phospholipid-binding proteins. Of note, patients in a subtype of APS develop multi-organ thromboses over the shortest periods (Catastrophic APS (CAPS)) like patients with severe COVID-19–associated coagulopathy. Both conditions show acute inflammatory response, cytokine storm, and highly elevated ferritin levels (179, 180). Furthermore, both conditions develop retiform purpura and livedoid rashes representing thrombotic microvascular injury with endothelial damage and cytokine reaction, spread by activation and deposition of complement that predisposes to thrombosis (181). However, thrombocytopenia is not as common as CAPS in COVID-19 (182). Therefore, there are clinical and laboratory similarities between severe COVID-19 and CAPS. However, we need more studies to delineate APL-related mechanisms of thrombosis in COVID-19 (178).

As aforementioned, males and females respond differently to MERS- and SARS-CoV infections in which the conditions are more threatening for males (183–185). Comparatively, there are important differences between men and women for cardiovascular diseases. To point out, men have a higher risk of first and recurrent venous thrombosis than do women (186). Considering COVID-19, different genetic and endocrine mechanisms might influence the mechanisms of coagulopathy and thrombosis in COVID-19 (187). Most compelling evidence shows genetic mechanisms of hemostasis do not differ in men and women. Henceforth, the greater risk of thromboembolism in men, even in the context of COVID-19, does not attribute to any sex-linked difference in genetic predisposition (186, 187). However, there are some pieces of evidence indicating the different role of endocrine mechanisms in regulating hemostasis in males and females. In humans, the plausible general rule is that under normal conditions of exposure to at least normal sex hormone levels, estrogens play a possible positive role and androgens play a negative role in hemostasis (187). Of note, testosterone deficiency in men correlated well with an increase in procoagulant factors and a decrease in anticoagulant factors contributes to the higher risk of thromboembolism in men than in women at any age including a greater frequency in men during elderly and a decreased frequency in women during fertile periods (188–192). To point out, the estrogen deficiency possibly explains why the incidence of thrombosis observed in the elderly is higher than in young women (187, 193). Given these points, a growing body of evidence seems to imply that deficiencies of sex hormones are a detrimental factor in the immune and inflammatory response or predisposition to thrombosis, upon which they present an unfavorable prognostic factor in the severity and outcome of COVID-19 (187, 194). To clarify, evidence imply that primary hypogonadism in males and secondary hypogonadism in both sexes may have a direct or indirect role in the development of systemic inflammation, endotheliopathy, and thromboembolism in patients with COVID-19 (129, 187, 194, 195). Altogether, pieces of evidence imply that the severity of COVID-19 deteriorates in older ages for both sexes, in men more than in women, possibly because of inflammaging and endothelial dysfunction with aging. However, it is not quite clear if sex hormones have different effects on immunity, inflammation, and thrombotic status, and if different reductions in sex hormones in both sexes affect the severity and outcome of COVID-19 (183–185, 187, 196, 197).

Besides susceptibility to age-dependent diseases, epidemiological studies indicated sexdifferences in incidence and Case Fatality Rates (CFRs) after SARS-CoV infection in humans in which males experience higher CFRs than females (183, 184). Similarly, data from recent MERS-CoV outbreaks showed high incidence and CFRs among men (185).

## Conclusion

COVID-19, especially in its severe form, is a multisystem syndrome with sex disparities in severity and outcome characterized by a state of immunothrombosis in which a complex interaction of SARS-CoV-2 virus invasion with platelets, leukocytes, endothelial cells, immune response, and the possible involvement of megakaryocytes resulting in endotheliopathy, coagulopathy and thromboinflammation that culminate in more severe complications and mortality. With this in mind, we may claim that the capability of interconnected biological levels of the organization is aberrated significantly by SARS-CoV-2 infection during COVID-19 progression, resulting in pathologically and fatally enhanced levels of crosstalks. A recent systematic review and meta-analysis of over 50 long-term effects of COVID-19 show that fatigue, headache, attention disorder, hair loss, and dyspnea are the five most common symptoms (198). Correspondingly, a more recent study (199) revealed that clotting markers elevated in patients with long COVID syndrome while inflammation markers had returned to normal, showing that the clotting system may be involved in the root cause of long COVID syndrome that helps to explain persistent symptoms such as reduced physical fitness and fatigue. To clarify, we propose that a deeper understanding of the coagulation and inflammation in acute COVID-19 may also be relevant for a better understanding of certain cases of long COVID.

## Author Contributions

All authors wrote the first draft of the manuscript. MEB and MN critically reviewed and edited the manuscript. ME and NA created figures. MEB supervised all aspects of the work. All authors contributed to the article and approved the submitted version.

## Funding

This project is supported in part by the Shahrekord university grant to MEB (grant number 1399).

## Conflict of Interest

MN was employed by Erythrogen Medical Genetics Lab.

The remaining authors declare that the research was conducted in the absence of any commercial or financial relationships that could be construed as a potential conflict of interest.

## Publisher’s Note

All claims expressed in this article are solely those of the authors and do not necessarily represent those of their affiliated organizations, or those of the publisher, the editors and the reviewers. Any product that may be evaluated in this article, or claim that may be made by its manufacturer, is not guaranteed or endorsed by the publisher.
